# Oral Health in Women with a History of High Gestational Diabetes Risk

**DOI:** 10.3390/dj7030092

**Published:** 2019-09-03

**Authors:** Hanna Poulsen, Jukka H. Meurman, Hannu Kautiainen, Anna Maria Heikkinen, Emilia Huvinen, Saila Koivusalo, Johan G. Eriksson

**Affiliations:** 1 Department of Oral and Maxillofacial Diseases and Helsinki University Hospital, University of Helsinki, PB 700, 00029 HUS, Finland; 2 Department of General Practice and Primary Health Care, University of Helsinki and Helsinki University Hospital, PO Box 20, 00014 Helsinki, Finland; 3 Department of General Practice and Primary Health Care, University of Eastern Finland, PO Box 1627, 70211 Kuopio, Finland; 4 Department of Obstetrics and Gynaecology, University of Helsinki and Helsinki University Hospital, PO Box 140, 00029 HUS, Helsinki, Finland; 5 Folkhälsan Research Center, University of Helsinki, PO Box 20, 00014 Helsinki, Finland; 6 Department of Obstetrics and Gynecology, National University Singapore, Yong Loo Lin School of Medicine, 1E Kent Ridge Road, Singapore 119228, Singapore

**Keywords:** gestational diabetes, oral health, periodontitis

## Abstract

We studied oral health in 115 women with and without a history of gestational diabetes (GDM), expecting poorer oral health in the GDM group. Full-mouth examinations were performed 5 years postpartum and the number of teeth, total dental index (TDI) and decayed, missing, filled teeth (DMFT) index were calculated. Bleeding on probing (BOP), probing depth (PD), visible plaque index (VPI), and clinical attachment level (CAL) were recorded. The periodontal inflammatory burden index (PIBI) was calculated. Panoramic radiographs were taken and signs of infections recorded. Oral health habits, symptoms and participants’ own opinion of oral health were recorded with questionnaires. At the time of examination, 45% of the women had a history of GDM in the index pregnancy. Mild periodontitis (62%) and bleeding on probing (46%) were common. VPI (13% and 17%, *p* = 0.009) and PIBI (13.1 and 17.5, *p* = 0.041) were lower among women with a history of GDM compared with those with no history of GDM. There was no difference between groups in DMFT scores. All women reported good subjective oral health. Thus, contrary to our hypothesis, women with a history of GDM showed better oral health parameters than women without a history of GDM.

## 1. Introduction

Poor oral health has been associated with metabolic disorders, including type 2 diabetes (T2D), and it might also affect GDM through low-grade systemic inflammation caused by chronic oral and dental infections [1]. Gestational diabetes mellitus (GDM) is defined as impaired glucose regulation during gestation and is diagnosed for the first time during pregnancy [2]. Since the incidence of GDM continues to rise, it is important to understand factors associated with this development. There are several known predisposing factors, such as obesity and increasing age, but probably many risk factors for GDM have not yet been identified.

Globally, GDM affects 1%–14% of pregnant women, and the incidence is increasing [1,3]. In 2017, the GDM incidence in Finland—where the present study was conducted—reached 19% of all pregnant women [4]. GDM is associated with complications both during and after pregnancy and it affects both the mother and the offspring [5,6,7]. GDM is a sign of an elevated risk for developing T2D, metabolic syndrome and cardiovascular disease [8,9,10,11,12]. Five years postpartum, the risk for T2D increases steeply and then continues to rise more steadily among women with a history of GDM. Women with GDM have a 7-fold risk of developing T2D compared to women with normoglycaemic pregnancies [8,13]. According to a Swedish study, 30% of women with a history of GDM were diagnosed with T2D 5 years postpartum and 51% had some form of impaired glucose regulation [14].

Obesity and being overweight, the most important risk factors for GDM, are associated with an increased risk for several inflammatory conditions and they can also cause unwanted pregnancy outcomes [13,15,16]. In 2017, according to the national Finnish Birth Register, 37.5% of women who gave birth were overweight (BMI > 25 kg/m^2^) before pregnancy. Besides obesity, advanced age, family history of diabetes, non-Caucasian ethnicity, parity, previous macrosomia and a history of GDM are risk factors for GDM [17].

Poor oral health has been linked to adverse pregnancy outcomes [18]. Associations between poor oral health, including dental caries and periodontitis, and being overweight have also been reported [19,20]. Abariga and Whitcomb suggested in their meta-analysis that periodontitis is associated with a significantly increased risk for GDM compared to women without periodontitis [21]. This chronic inflammatory condition induces local and systemic host immune responses and has been suggested to have a potential role in the development of GDM [21].

The effects of a lifestyle intervention among women at a high risk for GDM were evaluated in the Finnish Gestational Diabetes Prevention Study (RADIEL) trial [22]. In the present study, a sub-analysis of the RADIEL trial, we investigated whether poor oral health was associated with a history of GDM in women at high risk for GDM. Our study hypothesis was that oral infections such as periodontitis are linked to GDM and metabolic control, and therefore a history of GDM would be associated with markers of non-optimal oral health.

## 2. Materials and Methods

This study is a cross-sectional sub-study of the RADIEL GDM prevention trial (n = 720) described in detail by Rönö et al. [22]. Briefly, the Finnish Gestational Diabetes Prevention Study (RADIEL) is a randomized controlled multi-center intervention trial, set up to investigate the effect of a lifestyle intervention aimed at the prevention of GDM in women at high risk for GDM. Women in early pregnancy or women planning pregnancy with an increased risk for GDM due to having a BMI ≥ 30 kg/m^2^ or a history of GDM, were recruited to the study between the years 2008 and 2012. The participants were randomized into an intervention group, with intensified diet and exercise counselling, and a control group receiving usual care.

**Figure 1:**
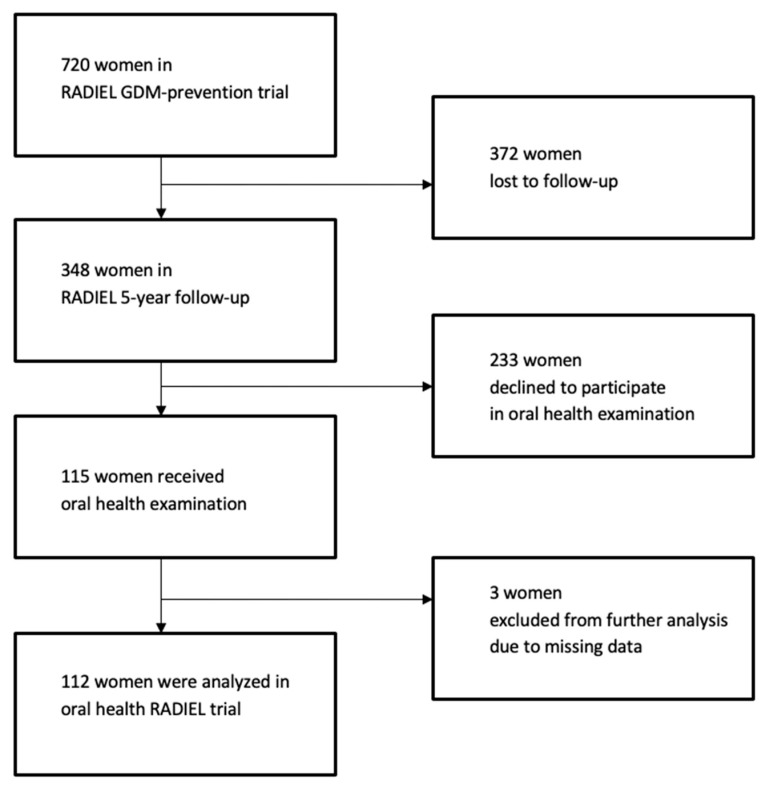
Study flow chart.

The present sub-study of RADIEL was a follow-up investigation including 115 women from the original cohort who were re-examined 5 years postpartum. The study flow chart is shown in Figure 1.

Maternal height and weight were measured. An electronic stadiometer was used to measure height to the nearest 0.1 cm, weight was assessed using an electronic scale to the nearest 0.1 kg. Body mass index (BMI) was calculated as weight (kg)/height^2^ (m^2^). Waist circumference was measured with a soft tape midway between the iliac crest and the lowest rib to the nearest 0.5 cm. Educational attainment, smoking habits and physical activity were assessed by validated questionnaires.

Blood samples were drawn by a trained laboratory technician after overnight fasting. Plasma glucose was determined using enzymatic hexokinase assay (Roche Diagnostics, Basel, Switzerland). Low density lipoprotein (LDL), high density lipoprotein (HDL), triglycerides (TG) and total cholesterol were determined using enzymatic assays. Glycated hemoglobin (HbA_1c_) was determined using immunoturbidimetric analyzer. A 2 h 75 g oral glucose tolerance test was performed for the assessment of glucose regulation.

The oral health examination included a full mouth examination and panoramic X-rays of the jaws. The examination was made by a single examiner (HP) who was unaware of the metabolic status of the participants. The number of teeth, dental and periodontal status, and mucosal health were recorded.

The visible plaque index (VPI) and bleeding on periodontal probing index (BOP) were calculated according to Ainamo and Bay [23]. Periodontal pocket depths (PDs) and the presence of calculus were recorded. A WHO probe was used to measure the variables from 6 surfaces of each tooth. Gingival recessions were measured from the enamel–dentin junction to the gingival margin. Periodontitis was defined as mild, moderate or severe according to Page & Eke [24]. According to this definition, in mild periodontitis, there are at least two interproximal sites with a clinical attachment loss (AL) of at least 3 mm and two or more interproximal sites with PDs of at least 4 mm or one site with a PD at least 5 mm. AL was measured as the difference between PD and GR. In moderate periodontitis, there are two or more interproximal sites with an AL of at least 4 mm or two or more sites with PDs of at least 5 mm. In severe periodontitis, there are two or more interproximal sites with an AL of 6 mm or more and one or more interproximal site with a PD of 5 mm or more. The interproximal sites are not located on the same tooth [24]. Decayed, missing, filled teeth (DMFT) index, total dental index (TDI), and periodontal inflammatory burden index scores (PIBI) were calculated [25,26].

From the panoramic X-rays, signs of infections in the apical regions of the teeth were recorded and marginal alveolar bone loss was measured. Oral health habits, possible symptoms and the participant’s own opinion of her oral health were recorded by using a structured questionnaire. 

All participants were informed about their clinical oral health and X-ray status. If the participant needed oral health care, she was advised to make an appointment to see a dentist. 

## 3. Statistical Analysis

The descriptive statistics are presented as means with SDs or as counts with percentages. Statistical comparison between the subjects with and without a history of GDM was performed by the t-test, Chi-square test, or Fisher–Freeman–Halton test when appropriate. Adjusted models, analysis of covariance (ANCOVA) and logistic models were used to compare the groups as oral health parameters. We fit three adjusted models in these analyses: Model 1 (adjusted for age), Model 2 (adjusted for age, smoking, fasting plasma glucose), and Model 3 (adjusted for age, smoking, fasting plasma glucose, tooth brushing frequency and education years). The bootstrap method was used when the theoretical distribution of the test statistics was unknown or in the case of violation of the assumptions (e.g., non-normality). Correlation coefficients between oral health parameters were calculated by the Spearman method, using Sidak-adjusted probabilities (significance level). The normality of the variables was tested by using the Shapiro–Wilk W test. The Stata 15.0 (StataCorp LP; College Station, TX, USA) statistical package was used for the analysis.

## 4. Ethical Approval

All participants gave their informed consent for inclusion before they participated in the study. The study was conducted in accordance with the Declaration of Helsinki, and the protocol was approved by the Ethics Committee of Women, Children and Psychiatrics of Helsinki University Hospital (136/13/03/03/2014) on 24 April 2014.

## 5. Results

Clinical characteristics of the study participants are given in Table 1. Almost half of the participants (44.6%) had GDM during the index pregnancy. Those women with a history of GDM had a higher fasting glucose and HbA_1c_ compared to women without a history of GDM during the index pregnancy. The prevalence of smoking was higher in women without a history of GDM. Four women, 2 with and 2 without a history of GDM, had developed T2D.

**Figure 2:**
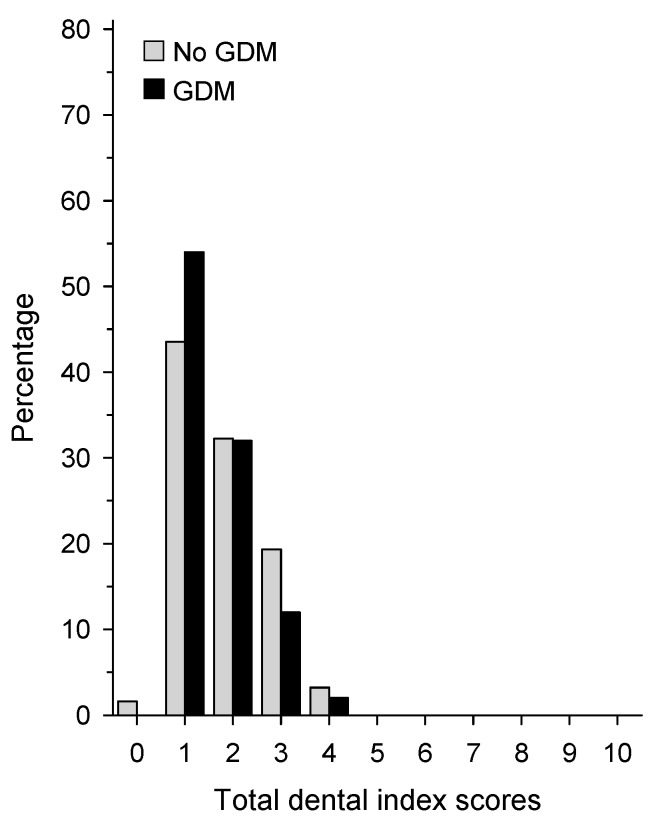
Total dental index scores (TDI) in women without a history of GDM (white bars) and with a history of GDM (dark bars).

The participants had an overall good oral health as assessed by the total dental index (TDI) score (Figure 2). The median number of teeth was 28 and only a few participants had lost more than 4 teeth (9%).

Of the women, 7 were diagnosed with periapical periodontitis and 6 with horizontal alveolar bone loss. Findings in the panoramic radiographs showed no difference between women with or without a history of GDM. In total, 10 women had mucosal findings including a small ulceration (one), leukoplakia (four) and redness (five women).

**Figure 3:**
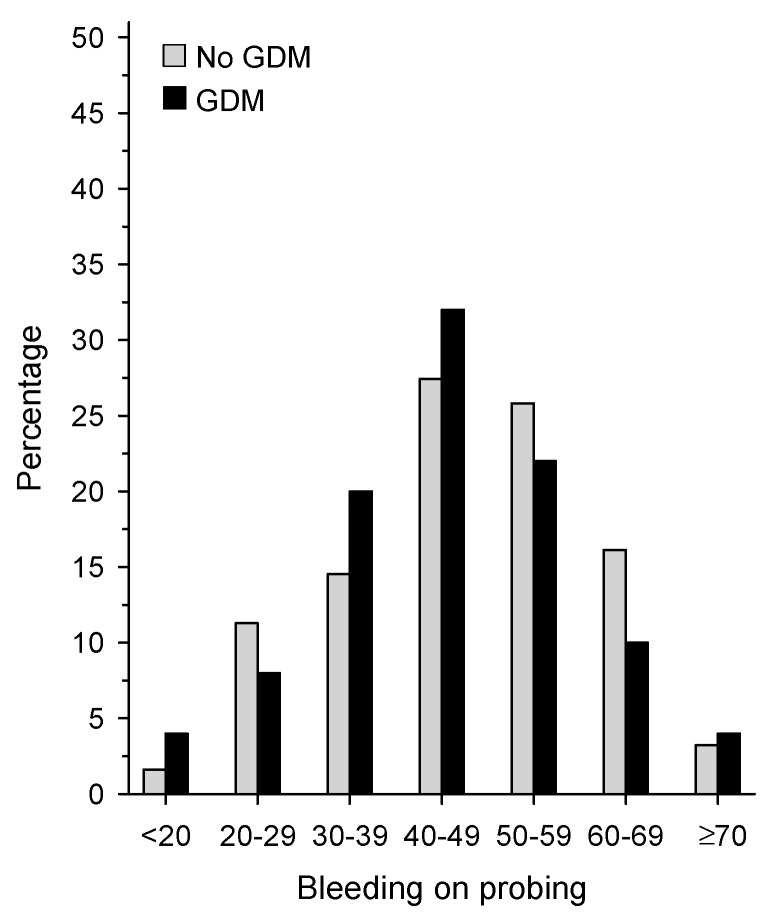
Bleeding on probing (BOP, %) in women without GDM (white bars) and with GDM (dark bars).

DMFT (9.80 and 8.92, *p* = 0.12), PIBI (13.1 and 17.5, *p* = 0.041) and VPI (13% and 17%, *p* = 0.009) scores were low, while PIBI showed a large variation. In this regard, the difference between women with and without a history of GDM was significant; those who did not have GDM had higher scores. Mild periodontitis was common in both groups, 62% (68% vs. 58%) of all participants, but women with no history of GDM had a higher prevalence of moderate or severe periodontitis than women with a history of GDM (22% vs. 32%) (Table 2). Women with no history of GDM had more dental plaque as reflected in the significantly higher VPI scores (Table 2). In all women, the BOP score was relatively high, with the mean of 46% (Figure 3).

Almost all women reported their oral health as good or moderately good. Only 4 participants reported poor oral health. Of the women, 60% (n = 68) reported unspecific subjective symptoms during the last 6 months. These were mainly mild discomfort and only one woman had dental pain.

Of the women, 76% reported brushing their teeth twice a day and 64% also used dental floss or inter-proximal brushes. Women with a history of GDM cleaned their interdental spaces more regularly (72%) than women with no history of GDM (58%) (Table 2).

The correlation analyses between the main oral parameters assessed are given in Table 3. The strongest correlations were observed between TDI and PIBI and between BOP and TDI (all *p* < 0.001).

## 6. Discussion

In this study we observed that gingivitis was prevalent among high-risk women for GDM when assessing their oral health 5 years postpartum. Severe periodontitis and other oral diseases were rare. The women had overall good oral health. No associations were found between the history of GDM and the oral health parameters assessed, 5 years after childbirth. Hence, our study hypothesis was not confirmed.

Periodontal disease is characterized by low-grade inflammation and it has been associated, among other things, with T2D, cardiovascular diseases and pregnancy complications [2,5,6,7,21,27,28,29]. Chronic infections cause low-grade systemic inflammatory reactions and, consequently, affect glucose metabolism with an increased risk for metabolic disorders [30]. Our study participants were all at an increased risk for GDM. Of them, 66% were overweight or obese and thus potentially subjected to low-grade inflammation with an increased risk for gingival bleeding and the development of periodontal disease. This, however, was not observed in the present study.

Better oral health habits, regular and thorough cleaning of teeth among women with a history of GDM might have been a consequence of motivation for better health care in general. Voluntary participation in the RADIEL trial may be a sign of the women’s interest in their health in general. The many study visits and received written information provided may indeed have encouraged them to take better care of themselves including their oral health habits. This may be the reason for the better oral health parameters observed among the women with a history of GDM.

A two-way relationship between periodontal disease and GDM has been reported [21,31]. However, the findings have been inconsistent and there is no strong evidence to support a direct association, nor causality [32]. An association between periodontitis and poverty and low educational attainment has also been reported [33]. Obesity and being overweight are also related to periodontitis, which is more frequent among women with a lower socioeconomic status [34]. In other words, it has been suggested that socioeconomical factors might be important factors which partly explain the association between GDM and poor oral health. However, the women who participated in the RADIEL trial had a high educational attainment, which might have positively influenced the study findings also in relation to their oral health. Further, the women in the RADIEL trial mostly had a mild type of GDM, since few needed drug therapy for their GDM. The majority of women diagnosed with GDM were diagnosed based on the fasting glucose value, and not the postprandial glucose values. This might also have an impact on oral health, since higher postprandial values have been associated with more oxidative stress and related inflammatory processes [35].

The strengths of this study are the unique data from women with high a risk of GDM who were studied from the beginning of their pregnancy, throughout pregnancy, and then re-studied 5 years postpartum. To our knowledge, this indeed is the first study on high-risk women for GDM assessing their oral health postpartum. Participants were from a homogeneous Caucasian population. The weaknesses of our study were that it was cross-sectional, and that we observed a large drop-out rate, which is common in clinical studies. Of all the women in the original RADIEL study (n = 720), only 48% participated in the 5-year follow-up examinations. Of these, 333 attended an oral glucose tolerance test and 34.5% (n = 115) participated in the oral examination reported here. Furthermore, oral health had not been examined at the time of pregnancy, which would have been useful for comparison to the 5-year postpartum results. Other limitations of this study were convenience sampling, statistical limitations, a lack of dietary data and the lack of measurement between oral diseases and a history of GDM. Hence, future research should focus on oral health of women at risk for GDM before, during and after pregnancy. In any case, however, maintaining good oral health throughout pregnancy should be important for preventing adverse health outcomes, both in the mother and her offspring [18,36].

## 7. Conclusions

To sum up, women with a history of GDM exhibited a slightly better oral health status compared with women without GDM history but at high GDM risk. Gingivitis nevertheless was prevalent, so prevention and treatment of oral diseases in women at risk for GDM needs to be emphasized.

## Figures and Tables

**Table 1:** Baseline characteristics of the study participants with and without a history of gestational diabetes (GDM) 5 years postpartum. IQR = interquartile range; CRP = C-reactive protein

	No History of GDM n = 62	History of GDM n = 50	*p*-Value
Age (years), mean (SD)	39 (6)	40 (4)	0.30
Weight (kg), mean (SD)	87.6 (15.8)	80.3 (19.9)	0.066
Height (cm), mean (SD)	167 (7)	166 (7)	0.52
BMI (kg/m^2^), mean (SD)	32.3 (6.0)	30.2 (7.6)	0.10
Waist circumference (cm), mean (SD)	105 (16)	102 (18)	0.36
Education years, mean (SD)	14.9 (1.8)	14.8 (2.0)	0.96
Smoking, n (%)	11 (18)	2 (4)	0.024
Physical activity, (min/week), median (IQR)	60 (30–160)	60 (35–135)	0.91
HbA_1c_, (%), mean (SD)	5.45 (0.30)	5.59 (0.30)	0.012
Fasting plasma glucose (mmol/L), mean (SD)	4.97 (0.46)	5.37 (0.58)	<0.001
Total cholesterol (mmol/L), mean (SD)	4.57 (0.74)	4.63 (0.74)	0.68
HDL cholesterol (mmol/L), mean (SD)	1.56 (0.35)	1.52 (0.41)	0.56
LDL cholesterol (mmol/L), mean (SD)	2.87 (0.71)	2.97 (0.68)	0.43
High-sensitivity CRP (mmol/L), mean (SD)	2.99 (4.88)	1.97 (2.55)	0.18
Impaired glucose regulation, n (%)	5 (8)	11 (22)	
Impaired fasting glucose, n (%)	1 (2)	4 (8)	
Impaired glucose tolerance, n (%)	2 (3)	5 (10)	
Diabetes mellitus, n (%)	2 (3)	2 (4)	

**Table 2:** Oral health parameters in women according to history of GDM.

	No GDM n = 62	GDM n = 50	*p*-Value			
			Crude	Adjusted		
				1.	2.	3.
Decayed, missing, filled teeth (DMFT), mean (SD)	8.92 (5.52)	9.80 (5.07)	0.40	0.61	0.12	0.10
Total dental index (TDI), mean (SD)	1.79 (0.89)	1.62 (0.78)	0.34	0.23	0.20	0.22
Periodontal inflammatory burden index (PIBI), mean (SD)	17.5 (13.3)	13.1 (10.5)	0.056	0.048	0.041	0.067
Bleeding on probing, mean % (SD)	47 (0.13)	45 (0.14)	0.34	0.31	0.22	0.28
Visible plaque index, mean % (SD)	17 (0.12)	13 (0.12)	0.11	0.089	0.009	
Periodontitis, n (%)			0.31	0.30	0.31	
no periodontitis	6 (10)	5 (10)				
mild periodontitis	36 (58)	34 (68)				
moderate or severe periodontitis	20 (32)	11 (22)				
Tooth brushing twice a day, n (%)	41 (66)	35 (70)	0.66	0.91	0.42	
Interdental cleaning, n (%)	36 (58)	36 (72)	0.13	0.17	0.20	

Adjusted 1: with age. Adjusted 2: with age, smoking, fasting plasma glucose. Adjusted 3: with age, smoking, fasting plasma glucose, toothbrushing frequency and education years.

**Table 3:** Results from correlation analyses between the oral health parameters.

	TDI	BOP	PIBI	VPI
DMFT	0.32 (0.14 to 0.48) **	−0.00 (−0.19 to 0.18)	0.12 (−0.06 to 0.30)	0.10 (−0.08 to 0.28)
VPI	0.15 (−0.03 to 0.33)	0.15 (−0.04 to 0.33)	0.31 (0.13 to 0.47) *	
PIBI	0.41 (0.24 to 0.55) ***	0.40 (0.23 to 0.54) ***		
BOP	0.17 (−0.02 to 0.31)			

Spearman correlations * 0.05, ** 0.01, *** 0.001; statistical significance calculated using Sidak-adjusted probabilities. TDI = total dental index; BOP = bleeding on probing; PIBI = periodontal inflammatory burden index; VPI = visible plaque index; DMFT = decayed, missing, filled teeth.
